# Editorial: Exploring brain connectivity to understand behavior

**DOI:** 10.3389/fnbeh.2023.1176953

**Published:** 2023-03-14

**Authors:** Gennady G. Knyazev

**Affiliations:** Laboratory of Psychophysiology of Individual Differences, Institute of Neuroscience and Medicine, Novosibirsk, Russia

**Keywords:** fMRI, connectivity, neuroimaging, behavior, resting state—fMRI

Since the seminal work of Biswal et al. (1995), the study of connectivity has occupied one of the most prominent places in neuroimaging, which is not surprising given the general understanding of its role in information transfer and implementation of brain functions. Beyond this general understanding, there are areas in which the study of connectivity has a distinct advantage over the study of activation. The realization of brain function at rest, when it is impossible to relate brain activity to the processing of specific stimuli, is one such area. However, there is reason to believe that the study of functional connectivity also has greater potential for identifying specific patterns associated with task performance than does the study of brain activation. One of the most difficult questions in neuroscience is how information is encoded in the brain. Numerous data accumulated by neuroimaging clearly show a lack of specificity in the task-related activation of most cortical areas. For example, Anderson and Penner-Wilger (2013) showed that the overall average diversity of different anatomical areas on a scale from 0 (active in only one cognitive area) to 1 (equally active in different cognitive areas) is 0.7. This diversity is much smaller for functional connections, which suggests that the specificity of information representation in the brain is provided by a task-specific pattern of connections rather than activations.

Most studies collected in this Research Topic use resting state fMRI (rs-fMRI) functional connectivity data. These data allow to investigate task-independent patterns of functional connections associated with pathological conditions or normal psychological processes. Herman et al., using independent component analysis to identify resting state networks, show that “decoupling” of the perceptual and somatosensory cortices, which can compromise effective integration of early perceptual information with behavioral control programs, may underlie impulsive behavior. Du et al. in their rs-fMRI study on a large sample of students show that task-independent spontaneous connectivity in the punishment network could explain the conformist tendency, which was measured using a conformity scale. Schienle et al. selected participants based on their responses in a survey about belief in miracles and showed that in people with high levels of belief, placebos can alter the experience of emotional salience and cognitive control, which is accompanied by connectivity changes in the associated brain networks. Xu et al. used rs-fMRI to reveal brain underpinning of the primary childhood emotional neglect (CEN) and found that college students with CEN history utilized the reappraisal strategy less frequently and displayed more depressive symptoms than a control group, which was accompanied by stronger prefrontal functional connections with other brain regions. Suo et al. recorded rs-fMRI in 122 earthquake survivors 10–15 months after the event and used connectome-based predictive modeling (CPM) to identify brain function features that are related to symptom severity. CPM predicted symptom severity scores based on functional connectivity between visual cortex, subcortical-cerebellum, limbic, and motor systems. The study highlights the potential usefulness of this kind of data for clinical assessment of PTSD symptom severity at the individual level.

Two studies explore changes in connectivity during experimental manipulations. Lee et al. using seed-based functional connectivity analysis of fMRI data show that autonomous sensory meridian response (ASMR), a sensory phenomenon in which audio-visual stimuli evoke a tingling sensation accompanied by a feeling of calm and relaxation, is accompanied by ongoing interaction between regions that mainly include mentalizing and self-referential networks. Contrary to the previous studies that used functional connectivity measures, Choi et al. employ effective connectivity measures in the framework of dynamic causal modeling (Friston, 2011). They show that simulated driving requires multi-domain executive function in addition to vision, and pathway activation is influenced by the driving experience and familiarity of the driver. Bari et al. use probabilistic tractography to evaluate whether structural connectivity of the amygdala to the brain reward network is associated with impulsive choice and tobacco smoking. Using data from the Human Connectome database, they analyze how subject performance on a delayed discounting task and whether they met specified criteria for difficulty quitting smoking could be predicted from sMRI measures. Findings highlight the importance of the amygdala-hippocampal-anterior cingulate network in the valuation of future rewards and substance dependence. Authors suggest that these results may help to identify potential targets for neuromodulatory therapies for addiction and related disorders. Finally, a study by Terstege et al. using Morris water maze training in mice and quantification of c-Fos-labeled fluorescent cells examines the effect of prior chronic spatial training on task-specific functional connectivity associated with subsequent contextual fear recall. Results show an increase in global efficiency and in network resilience based on simulated targeted node deletion. Overall, this study suggests that chronic learning has transferable effects on the functional connectivity networks of other types of learning and memory. The generalized enhancements in network efficiency and resilience suggest that learning itself may protect brain networks against deterioration.

To summarize, the collection of articles in this Research Topic demonstrates the variety of areas of neuroscience in which the study of connectivity can yield exciting discoveries. They range from elucidating the cerebral underpinnings of persistent individual differences, such as impulsivity, conformity, and suggestibility, to the short-term or long-term effects of experimental manipulations, such as ASMR, simulated driving, and water maze training, or environmental events and conditions, such as earthquakes or CEN. Even more exciting discoveries can be expected in the near future, when recently developed methods such as dynamic connectivity or multivariate pattern analysis breathe new life into the study of connectivity.

## Author contributions

The author confirms being the sole contributor of this work and has approved it for publication.
